# Understanding drinking among midlife men in the United Kingdom: A systematic review of qualitative studies

**DOI:** 10.1016/j.abrep.2018.08.001

**Published:** 2018-08-04

**Authors:** Hannah Parke, Monika Michalska, Andrew Russell, Antony C. Moss, Clare Holdsworth, Jonathan Ling, John Larsen

**Affiliations:** a Drinkaware, London, United Kingdom; b Centre for Addictive Behaviours Research, London South Bank University, London, United Kingdom; c School of Applied Sciences, London South Bank University, London, United Kingdom; d School of Physical and Geographical Sciences, Keele University, Keele, United Kingdom; e Department of Pharmacy, Health and Wellbeing, University of Sunderland, Sunderland, United Kingdom

**Keywords:** United Kingdom, Systematic review, Thematic synthesis, Alcohol, Midlife men, Experience, Sociocultural meaning

## Abstract

**Objectives:**

This study reviews qualitative research into the sociocultural meanings and subjective experiences that midlife men in the United Kingdom (UK) associate with their drinking. In the UK, average weekly alcohol consumption is highest among midlife men, and they are disproportionately affected by alcohol harm. There is increasing recognition that public health messages to support behaviour change must be based on an in-depth understanding of drinking motivations and experiences.

**Study design and methods:**

Systematic literature review of studies exploring motivations for and experiences of drinking among UK men aged 45–60 using qualitative methodology. Medline, PsycINFO and the Social Science Citation Index were used, along with manual searches of key journals, Google searches and a call for evidence. The Critical Appraisal Skills Programme tool was used to quality-assess papers. Thematic synthesis was used to combine and analyse the data.

**Results:**

From 5172 titles and abstracts (1995–2018), 11 publications were included, representing 6 unique studies. Five themes were identified: ‘Drinking Motivations’; ‘Drinking Justifications’; ‘Drinking Strategies and Control’; ‘Social Norms and Identity’ and ‘Harm’. Motivations for drinking among midlife men were associated with relaxation, socialising and maintenance of male friendships. They justified drinking as a choice and emphasised their ability to meet responsibilities, which they contrasted with ‘problem drinkers’. Social norms governed drinking behaviours as an expression of masculinity.

**Conclusion:**

This review highlights the significance of the meanings and social importance of alcohol consumption among midlife men. Interventions using information and guidance should consider these when aiming to effectively influence the way this group drinks.

## Highlights

• Motivations for drinking among midlife men were associated with relaxation, socialising and maintenance of male friendships.
• Midlife men saw their drinking as a choice and in contrast to ‘problem drinkers’ who cannot meet key responsibilities.
• Strong social norms govern drinking amongst this group, with alcohol consumption seen as an expression of masculinity.
• There is an evidence gap of primary studies focussing on drinking in middle-aged men.

## 1 Introduction

Alcohol consumption globally represents the fifth largest single cause of premature mortality, loss of health and disability (Lim et al., 2012) and in the United Kingdom (UK), rates of alcohol-related deaths remain higher than 20 years ago (Office of National Statistics, 2015). In England and Scotland middle-aged men have the highest average weekly alcohol consumption (McLean, Christie, & Gray, 2017; NHS Digital, 2016), and men are disproportionately affected by alcohol harm. In the UK there were 8758 alcohol-related deaths in 2015, and two-thirds (65%) of these were male. Research, policy and media attention has tended to focus on the deliberate, hedonistic pursuit of intoxication (‘extreme drinking’) among young people in the night-time economy (Christmas & Seymour, 2014; Martinic & Measham, 2008), and less attention is directed towards the cumulative harms from drinking, although alcohol-related death rates in the UK in 2015 were highest among men aged 55 to 69 years (42.2–44.9 deaths per 100,000 population) (Office of National Statistics, 2015). Specifically, the 45–64 age group contains a quarter of the population but account for a half of all alcohol-related deaths (Erskine et al., 2010).

Increased alcohol consumption and alcohol-related problems among older age groups in many developed countries (Hallgren, Högberg, & Andréasson, 2010; Veenstra & Syse, 2012) mean that there is now increasing awareness of the need to better understand drinking among middle-aged and older people (Wilson et al., 2013). The link between amounts of alcohol consumed and health risks has been highlighted in the evidence informing the new UK alcohol guidelines (Department of Health, 2016), and, in contrast to some previous perceptions, drinking in later life should be recognised not as a cause, but an indicator, of good health (Holdsworth et al., 2016). Among individuals aged 55–65 years the risk of mortality over 20 years is increased by 23% for those consuming a daily average of 1–3 standard drinks (14–42 g alcohol) and by 70% for those drinking at least 3 drinks daily (Holahan et al., 2010). An important consideration is the observation of disproportionate harm in lower socioeconomic groups (Erskine et al., 2010), regardless of a lack of evidence of higher levels of consumption (Beard et al., 2015). A likely explanation of this ‘alcohol harm paradox’ is the multiplying risk impact from the combination of wider lifestyle factors related to smoking, diet and lack of exercise more commonly found in lower socio-economic groups (Bellis et al., 2016).

Initiatives to reduce alcohol-related harm may include a range of tools to affect the key drivers of affordability, availability and acceptability (Burton et al., 2017), and efforts directed at changing people's drinking behaviour should consider their capabilities, opportunities and motivations for making such changes (Michie, van Stralen, & West, 2011; Michie et al., 2012). Public health information campaigns to reduce alcohol consumption are only one aspect of such wider efforts, and they have traditionally aimed to encourage reasoned decisions about health (Heather, 2011). However, public health campaigns focussing on health risks have limited impact where the risk to health is from a consumable substance which can be perceived as desirable, such as alcohol, and where health risks are not immediate (Frankel, Davison, & Smith, 1991). Hence, it is not surprising that public health information campaigns conceptualising alcohol consumption as an individual behaviour resulting from rational choice have been found to be relatively ineffective (Babor et al., 2010).

However, health information campaigns aiming to reduce alcohol consumption may fail to capture the meanings and the context of drinking (Lyons, Emslie, & Hunt, 2014). Campaigns frequently focus on increasing knowledge of a particular behaviour (for example, a recommended number of standard alcohol units) and assume that people will choose to amend their drinking in line with recommendations. This approach wrongly assumes that individuals are primarily rational beings whose behaviour is devoid of social context or social meaning (Backett & Davison, 1995) and it is unable to predict or change behaviour (Mielewczyk & Willig, 2007). It has been highlighted as a limitation that alcohol harm reduction public health campaigns typically focus on alcohol unit measurement guidelines, and are not considering acceptable drinking practices among the target audiences (Thurnell-Read, 2017). In order to engage the public effectively it is important to understand the meanings and values people ascribe to their drinking, and the ‘lay epidemiology’ used to explain the impact of drinking (Lovatt et al., 2015). How the consumption of alcohol is experienced and understood is inevitably social, cultural and gendered (Lyons et al., 2014), and it is critical to acknowledge that drinking practices and experiences are diverse and heterogeneous (Jayne, Valentine, & Holloway, 2010). This is increasingly being recognised beyond a tradition of research on the subject in the social sciences (Castro et al., 2014).

A survey of a UK representative sample of adults sought to capture such insights into drinking attitudes and behaviours (IPSOS Mori, 2015). Based on these findings, drinkers were segmented according to their attitudes and values (openness to moderation, reasons for drinking, mental wellbeing) and their behaviours (risk level of drinking, consequences and harms experienced from drinking). Five clusters were identified: ‘comfortable social drinkers’, ‘controlled home drinkers’, ‘risky social and coping drinkers’, ‘self-contained moderate drinkers’ and ‘risky career drinkers’. While the ‘risky social and coping drinkers’ broadly fitted with a profile of younger adults drinking excessively on nights out, the ‘risky career drinkers’ stood out by being predominantly male, over 45 years old and unlikely to moderate their drinking. Using the Drinking Motive Questionnaire, DMQ-R SF (Kuntsche & Kuntsche, 2009) this group was found to be drinking frequently for social and enhancement reasons (e.g., to have fun and to get drunk), although there was also evidence of drinking for coping reasons (e.g., to alleviate personal problems and worries). While the segmentation analysis provided a useful overview of UK adult drinkers and together with the epidemiological harm data presented a strong case for targeting midlife men in an effort to reduce their drinking, further in-depth insights are needed.

This paper sets out to systematically review existing qualitative research into the sociocultural meanings and subjective experiences that midlife men in the UK associate with their drinking, in order to inform efforts to encourage these men to reduce their drinking to less harmful levels.

## 2 Methods

### 2.1 Literature searching

Database searches were conducted in Medline, PsycINFO and the Social Science Citation Index from January 1995 to April 2018, restricted to those published in English. Search terms included “alcohol*”, “drunk*”, “motivat*”, “behav*”, and “attitud*” as well as MeSH terms where appropriate. Terms were combined using “AND” or “OR”. See S1 for full search strategy. Google Scholar was searched in May 2018, and key journals on alcohol consumption were searched manually: *Addiction*, *Alcohol and Alcoholism*, and *Addictive Behaviours*. A call for evidence was sent to experts in the field and issued on the Drinkaware (a UK alcohol harm reduction charity) website. For all included studies, a forward citation search was conducted using ISI Proceedings (Web of Science). The reference lists of all included publications were screened.

### 2.2 Study selection

Study selection involved two phases. Based on title and abstract papers were excluded if they did not explore motivations for, or experiences with, alcohol consumption; exploration of drinking motivations or experiences was required to be either an aim of the study, or a substantial finding in the results. To ensure an in-depth understanding and rich description of motivations and experiences only studies presenting primary data using qualitative methods were included. Participants were required to include midlife men (aged 45 to 60) living in the UK, to ensure their experiences and views were meaningfully captured as relevant to the specific life course and sociocultural context (e.g. qualitative insights are not likely to be meaningfully compared if pertaining to different age groups or diverse cultural traditions). Studies were excluded if published in a non-English language, prior to 1995, or including a sample of exclusively lifetime abstainers, or people with a past or present alcohol addiction. These exclusion criteria were introduced in order to ensure that experiences and views were current (as attitudes and behaviours may change over time) and relevant to the defined population (e.g. a person with a clinical diagnosis of alcohol addiction is likely to have different experiences than a person who does not). After full text screening papers were excluded if findings could not be identified as relating either specifically to midlife men or be relevant across age and gender groups.

Where different publications from the same research were identified (e.g. journal paper and full study report), either the most recent or the version with the focus most closely aligned to the aims of this review was included.

An initial screening was undertaken of papers published January 1995 to July 2015. 10% of titles and abstracts were screened by two reviewers (HP and MM), with an inter-rater agreement of 98%. Consensus was established through discussions with co-authors (JLa and AR). The remaining screening was conducted by one reviewer (HP or MM). A second screening was undertaken of papers published August 2015 to April 2018, and again 10% of titles and abstracts were screened by two reviewers (ACM and JLa), with an inter-rater agreement of 97%, and the remaining screening conducted by one reviewer (ACM). All full-text papers were screened independently by two reviewers (HP and MM, or ACM and JLa). Inter-rater agreement was 88%; in the 10 instances of disagreement a third reviewer (JLa or AR) acted as arbitrator.

### 2.3 Quality assessment and data extraction

The quality of included studies was assessed using the Critical Appraisal Skills Programme (CASP) criteria for qualitative studies (Critical Appraisal Skills Programme, 2013). For each of the 10 quality questions, a paper could be awarded 2 points if the criterion was fully met, 1 point if partially met, and 0 points if not met at all. This provides a maximum quality score of 20. See S2 for the operationalised CASP quality criteria. The quality of all studies was appraised by one study author (HP), with a sample appraised by a second study author (JLa) to check consistency (Campbell et al., 2003; Munro et al., 2007).

Data were extracted from the papers using a purpose-built extraction table. Data extraction was performed by one review author (MM) and checked by a second (HP). The findings of each study was taken to be all text under the heading ‘findings’ or ‘results’, including, but not limited to, illustrative quotes (Thomas & Harden, 2008). See S3 for full details of the data extraction table.

### 2.4 Data synthesis and analysis

Thematic synthesis was used to combine and analyse the data (Thomas & Harden, 2008). The particular value of qualitative research is the rich detail and contextualisation it offers regarding a particular setting or group of individuals; and the method of thematic synthesis provides an analytical process for bringing together such insights from a defined empirical area of study. As described by Pearce et al. (2015), a review of qualitative studies involves three levels of interpretation. Primary study participants, who interpret their own experiences when discussing them in interviews or focus groups, perform the first level of interpretation. The primary study authors, who analyse and interpret the data collected from research participants, perform the second level of interpretation. The third level of interpretation involves the synthesis of all the findings from the primary research studies. It is this third level of interpretation that this review aims to perform.

The first step of the process of thematic synthesis was line by line coding of the data. Free codes were applied, with in vivo codes used wherever possible. The reviewer (MM) adopted an iterative approach, continually adding to the bank of codes, revisiting old codes, and merging or revising existing codes where necessary. A second reviewer (HP) coded a sample of the data (n = 9), providing a second check and constructively challenging and questioning codes (Barbour, 2001). The final free codes and their organisation into descriptive themes were considered (HP, MM, AR, JLa); and codes and sub-themes were re-examined and refined through further group discussion. All authors contributed to the final analysis and write-up.

## 3 Results

**Fig. 1:**
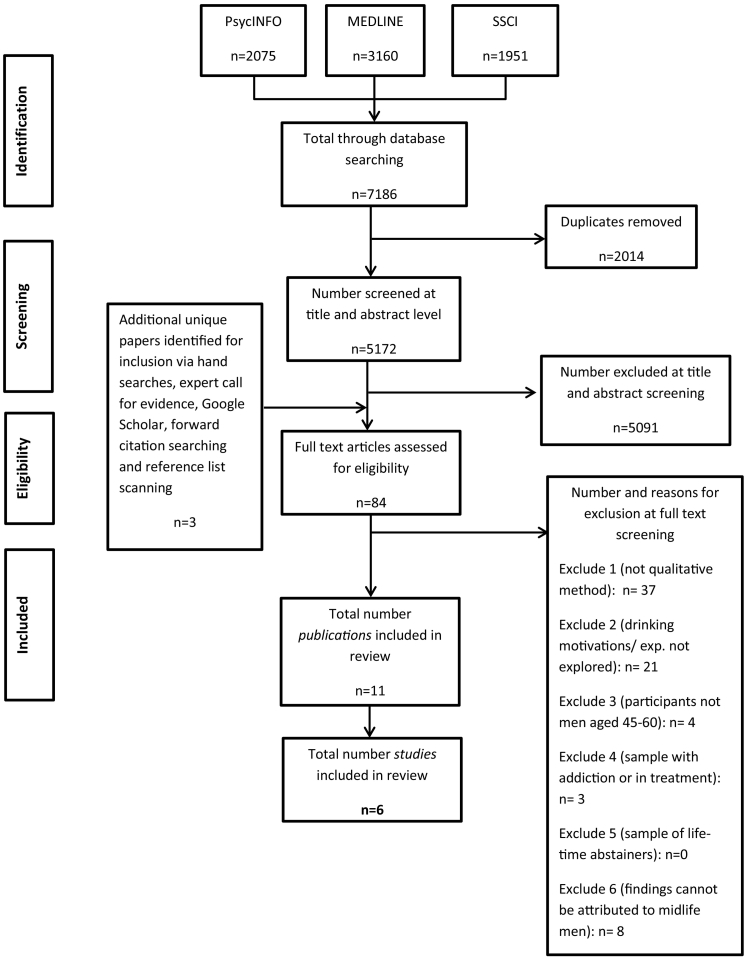
PRISMA flow chart.

The database searches produced a total of 5172 results after duplicates were removed. 5091 were excluded after title and abstract screening and additional three unique papers were identified for inclusion via hand searches, expert call for evidence, Google Scholar, forward citation searching and reference list scanning, leaving 84 papers to be screened in full. After full text screening 73 papers were found not to meet the inclusion criteria, leaving 11 publications included in the review. See Fig. 1 for the PRISMA flowchart of numbers.

### 3.1 Summary of included papers

The 11 publications were published between 2002 and 2014, and represent six unique studies. The studies took place across England and Scotland, with two unique studies conducted in the North East of England (Brierley-Jones et al., 2014; Wilson et al., 2013): one unique study in the West of Scotland (Emslie, Hunt, & Lyons, 2012; Emslie, Hunt, & Lyons, 2013; Lyons et al., 2014); one study in Blackpool (Foster & Heyman, 2013; Foster et al., 2010); one in Birmingham (Orford et al., 2002; Orford et al., 2009; Rolfe et al., 2006); and one in the South of the UK (Ritchie, 2007).

Only one publication included a study population of exclusively men in middle age (defined by study authors as 30 to 50) (Emslie et al., 2013), two other publications from the same study explored drinking in midlife for both men and women (Emslie et al., 2012; Lyons et al., 2014). The remaining publications included both men and women of different ages. One paper included participants aged 50 or over (Wilson et al., 2013); three publications from the same study started with a cohort of participants aged 22–50 and followed them over a 10-year period (Orford et al., 2002; Orford et al., 2009; Rolfe et al., 2006); another paper included participants aged 21–55 (Brierley-Jones et al., 2014); the remaining three publications included a wide range of ages (Foster & Heyman, 2013; Foster et al., 2010; Ritchie, 2007). Quotes from the papers illustrating their relevance to middle-aged men are presented in S4.

One unique study stated that participants were socioeconomically diverse (Emslie et al., 2012; Emslie et al., 2013; Lyons et al., 2014), and another unique study had sought optimum distribution by socioeconomic status and employment status (Orford et al., 2002; Orford et al., 2009; Rolfe et al., 2006). One study recruited only professional, managerial or clerical employees (Brierley-Jones et al., 2014). The remaining three unique studies did not provide information on socioeconomic or employment status.

Three unique studies provided information on the alcohol consumption of participants. Using the UK definitions in place at the time, one study included drinkers classified as low risk (<14 (female) or 21 (male) units/week, with one UK alcohol unit defined as 8 g pure alcohol), hazardous (>14 or >21 units/week), or harmful (>35 or >50 units/week) (Emslie et al., 2012; Emslie et al., 2013; Lyons et al., 2014). In another study all participants were classified as harmful drinkers (Orford et al., 2002; Orford et al., 2009; Rolfe et al., 2006). Lastly, Wilson and colleagues describe a range of drinking habits among their participants, from ‘occasional’ or ‘sensible’ drinkers, to those who were abstinent or dependent (Wilson et al., 2013).

Ethnicity was addressed in two unique studies: one only included White participants (Emslie et al., 2012; Emslie et al., 2013; Lyons et al., 2014) and another presented an ethnically diverse sample (Orford et al., 2002; Orford et al., 2009; Rolfe et al., 2006). The remaining studies did not state the ethnicity of participants.

Six publications used focus group methodology (Brierley-Jones et al., 2014; Emslie et al., 2012; Emslie et al., 2013; Foster & Heyman, 2013; Foster et al., 2010; Lyons et al., 2014); three employed interview methodology (Orford et al., 2002; Orford et al., 2009; Rolfe et al., 2006); and two used both focus group and interview methodology (Ritchie, 2007; Wilson et al., 2013). See Table 1 for a summary of the included publications.

**Table 1:** Details of included publications (n = 11).

Authors and date	Focus of research	Study inclusion criteria and recruitment methods	Sample	Method of data collection
Brierley-Jones et al. (2014)	Attitudes, meanings, and reported behaviour in relation to alcohol consumption.	• Professional, managerial and clerical employees who worked full-time. • Recruited by internally circulated email within workplaces in the North-East of England.	• 49 participants aged 21–55. Employment status ranged from junior to senior. Living in the North-East of England. • Alcohol consumption data not reported.	• Focus groups (n = 5) naturally occurring within workplaces. 4 mixed sex groups; 1 female-only. • Year of data collection not reported.
Emslie, Hunt, and Lyons (2012) [DrAM 1 of 3]	How men and women in early midlife perceive drinking and excessive alcohol consumption.	• Men and women aged 30–50 who regularly drank alcohol. • Recruited by: approaching people on street or in bars; contacting community groups and workplaces; advertising on community websites and snowballing.	• Sub-sample of 36 participants (15 m, 21 f) in midlife (aged 30–50). All White, living in the West of Scotland, socioeconomically diverse. • 17 low risk drinkers (<14 or <21 units/week); 13 hazardous drinkers (>14 or >21 units/week); 6 harmful drinkers (>35 or >50 units/week).	• Focus groups (n = 8) naturally occurring. 5 mixed sex groups; 1 male-only; 2 female-only. • Conducted 2009.
Emslie, Hunt, and Lyons (2013) [DrAM 2 of 3]	How men in midlife represent their alcohol consumption and how cultural construction of gender influences drinking.		• Sub-sample of 22 men aged 28–52. All White, living in the West of Scotland, socioeconomically diverse. • 6 low risk drinkers (<21/week); 12 hazardous drinkers (>21 units/week); 4 harmful drinkers (>50 units/week).	• Focus groups (n = 9) naturally occurring; 6 mixed sex groups; 3 male-only. • Conducted 2009–2011.
Lyons, Emslie, and Hunt (2014) [DrAM 3 of 3]	The relationships between embodiment, emotions, and alcohol, drinking, and drinking practices.		• Sub-sample of 56 participants (22 m, 34 f) in midlife (aged 30–50) most in their 30s and 40s. All White, living in the West of Scotland, socioeconomically diverse. • 2 reported no alcohol consumption in previous week; 22 low risk (<14 or <21 units/week) 32 hazardous drinkers (>14 or >21 units/week).	• Focus groups (n = 14) naturally occurring colleagues or friends: 6 mixed sex groups; 3 male-only; 5 female-only. • Conducted 2009–2011.
Foster, Read, Karunthi et al. (2010) [1 of 2]	Why people drink outside licensed premises.	• Both genders and differing age bands. • Recruited through Blackpool based voluntary sector organisations or residents groups.	• 38 participants (17 m, 21 f), all living in Blackpool. • Alcohol consumption data not reported.	• Focus groups (n = 4) naturally occurring. All mixed sex groups. • Conducted autumn/winter 2008.
Foster and Heyman (2013) [2 of 2]	How people drinking outside licensed premises think about the time framing of risks associated with alcohol consumption.			
Orford, Dalton, Hartney et al. (2002) [BUHD 1 of 3]	How excessive drinking is maintained.	• Men and women drinking >50 units (men) or 35 units (women) a week, aged 22–55, and untreated for any drinking problems within the last 10 years. Optimum distribution by sex, socioeconomic status, employment status and ethnic group. • Recruited through: adverts in newspapers, shop windows and on buses; hand delivered letters; leaflets; snowballing.	• Sub-sample of 50 participants (74% m, 26% f) aged 25–55, all living or working in the West Midlands. • All harmful drinkers (>35 units, or >50 units/week)	• (Qualitative data only) one-to-one semi structured interviews (n = 50). • Interviews conducted 1997.
Rolfe, Dalton, Krishnan et al. (2006) [BUHD 2 of 3]	The relationship between heavy drinking and aggression.		• Sub-sample of participants, with whom the following topics were included in the interview schedule (NB these are not necessarily mutually exclusive) Alcohol & aggression (n = 52) Domestic violence & aggression (n = 7) Gender and alcohol (n = 49) Masculinity and alcohol (men only, n = 23) Femininity and alcohol (women only, n = 25) • All living or working in the West Midlands. • All harmful drinkers (>35 units, or >50 units/week)	• (Qualitative data only) one-to-one semi structured interviews. • Interviews conducted 1999.
Orford, Rolfe, Dalton et al. (2009) [BUHD 3 of 3]	Places of drinking in the community and the functions these places serve.		• Sub-sample of 79 participants, all living or working in the West Midlands. • All harmful drinkers (>35 units, or >50 units a week)	• One-to-one semi structured interviews. • Interviews conducted 2003.
Ritchie (2007)	The consumption behaviour of moderate, social UK wine consumers.	• Wine consumers with age and gender balance. • Recruited through snowballing techniques and wine tasting group (‘expert’ group only)	• 43 participants aged 18 to over 55 living in the South of the UK. • 1 focus group with ‘expert’ ‘wine buffs’. • Alcohol consumption data not reported.	• Focus groups (n = 6). 4 mixed sex, 1 all-male, 1 all-female. Informed by one-to-one semi structured interviews used to develop question schedule. • Focus groups conducted March & April 2005.
Wilson, Kaner, Crosland et al. (2013)	Older people's reasoning for drinking in later life and how this interacts with health concerns.	• Both genders, broad range self-reported drinking, aged 50+. • Recruited through national charity for older people and services for alcohol problems.	• 24 participants (12 m, 12 f) aged 51–90 in interviews. • 27 participants (6 m, 21 f) aged 50–95 in focus groups. • All participants from North East England. • Range of drinking- occasional, ‘sensible’, abstinent and dependent.	• In-depth interviews. • Focus groups (n = 3). • Data collection in 2010.

The quality of the publications based on the CASP criteria ranged from 18 (Wilson et al., 2013) to 13 (Foster & Heyman, 2013; Ritchie, 2007) out of a maximum possible score of 20. See S5 for details of the quality appraisal scores for the included publications.

### 3.2 Analysis

Five themes were identified: ‘Drinking Motivations’; ‘Drinking Justifications’; ‘Drinking Strategies and Control’; ‘Social Norms and Identity’ and ‘Harm’. These will now each be discussed in turn. See S6 for illustrative quotes of each sub-theme identified.

### 3.3 Drinking motivations

This theme explores the reasons participants gave for drinking. The sub-themes identified were: drinking to relax and its distinction from drinking to cope; drinking to socialise and its importance in establishing and maintaining friendships; and drinking to get drunk.

Drinking was widely described as a means of relaxing and unwinding (Brierley-Jones et al., 2014; Emslie et al., 2012; Lyons et al., 2014; Orford et al., 2002; Wilson et al., 2013). For some, consuming alcohol was a way of distinguishing between the everyday practices of work, childcare, or household chores, and rest time. Alcohol was associated with a sense of freedom and escape from everyday routines (Brierley-Jones et al., 2014; Lyons et al., 2014; Orford et al., 2002). The distinction between drinking to relax and drinking to cope was often blurred by participants. Orford notes an ambiguity in the language used by participants, which allowed the coping function of alcohol to often remain hidden, with terms such as ‘unwind’ or ‘relax’ used more frequently (Orford et al., 2002). However, some explicit mentions of alcohol to facilitate coping were identified, for example coping with chronic pain (Wilson et al., 2013) or unemployment (Orford et al., 2002). Drinking outside the home in pubs or bars was seen as an important source of social support, a way for men to share their problems, seek advice, and maintain mental wellbeing (Emslie et al., 2012; Orford et al., 2009).

For many men, drinking was the central focus on which many friendships and relationships were established and maintained. Drinking was widely regarded as a highly sociable activity associated with having fun and bonding with others. Examples of the perceived positive, sharing nature of drinking included splitting a bottle of wine over dinner at home (Ritchie, 2007), buying rounds in the pub (Emslie et al., 2013), the pub as a community to which drinkers could belong (Orford et al., 2009) and the creation of drinking stories contributing towards a group's shared identity (Emslie et al., 2012). Importantly, many men perceived limited, or no, alternative options for socialising other than drinking together. Emslie found male participants laughed at the idea of going for lunch, or for a coffee, with male friends; for them the pub was essential for seeing friends and socialising (Emslie et al., 2013).

Drinking with the intention of getting drunk was also identified in a number of studies (Brierley-Jones et al., 2014; Emslie et al., 2012; Foster & Heyman, 2013; Lyons et al., 2014). Some reported a need to feel out of control, or to let off steam, and getting drunk was a means of achieving this. Additionally, some participants identified that certain forms of drinking contributed to a sense of self-esteem or image. This is further explored within the ‘Social Norms and Identity’ theme.

### 3.4 Drinking justifications

A common theme was the importance of justifying alcohol consumption. Three key sub-themes were: drinking as a controlled choice; meeting responsibilities; and ‘othering’ of problematic drinking as something unrelated to own identity.

Drinking was presented as something done out of choice. Participants emphasised that they were able to control and restrain their drinking, and in this way asserted that their alcohol consumption was unproblematic (Brierley-Jones et al., 2014; Foster & Heyman, 2013; Wilson et al., 2013). Some midlife men justified their excessive drinking by describing the increased tolerance to alcohol which they had built up over the years (Wilson et al., 2013). Drinking habits were justified by asserting that alcohol would never compromise their ability to meet their responsibilities. Participants presented themselves as responsible drinkers who arranged their drinking around key duties (namely childcare, employment, and driving). This included restricting drinking until later in the evening once children were in bed, and limiting drinking to the weekends due to concerns over driving to work and professional performance (Brierley-Jones et al., 2014; Emslie et al., 2012; Foster & Heyman, 2013; Lyons et al., 2014).

Commonly, participants talked about an ‘other’ kind of drinker whom was a cause for concern, contrasting this ‘problematic other’ to their own, unproblematic drinking. Young drinkers were characterised as less experienced and with lower alcohol tolerance. Their drinking was associated with an explicit aim of getting drunk quickly, increased aggression and causing public nuisance. This youthful drinking was contrasted with the ‘civilised’ drinking of the middle aged (Emslie et al., 2012; Foster et al., 2010; Rolfe et al., 2006). Participants reflected on how their drinking had changed over time, transitioning from the problematic youthful drinker to the respectable and civilised middle aged drinker. However, Emslie notes that while the dominant discourse was one of ‘older and wiser’, participant narratives challenged this in ways related to the following theme ‘Drinking Strategies and Control’. (Emslie et al., 2012).

### 3.5 Drinking strategies and control

Participants described the strategies they used to reach an optimal state of intoxication, how it was achieved and the consequences associated with failure to achieve it. The following sub-themes were identified: drinking strategies; being ‘in the zone’; knowing when to stop; drinking more with age and feeling superior to younger men; passing the ‘point of no return’; and loss of control.

Some participants used particular drinking strategies to enhance their drinking experience and reduce negative consequences of excessive drinking. These included eating certain foods to moderate the effects of alcohol and reduce the likelihood of making oneself vomit so as to ‘keep drinking’ (Lyons et al., 2014). In addition, some midlife drinkers described following certain drinking patterns and choosing particular types of alcohol in order to achieve desired psychological effects (e.g. drinking beer was associated with ‘hilarity’ (Brierley-Jones et al., 2014)). This links to participants reporting differences in physical and psychological effects arising from the type of alcohol they consumed, especially when trying a new unknown brand or substituting beer for wine (Lyons et al., 2014) which was associated with unpredictable effects (Rolfe et al., 2006).

The second sub-theme of being ‘in the zone’ relates to a pre-planned, optimal, intoxicated state which participants associated with enjoyment and used to justify their drunkenness. The participants expressed their desire to reach this state and revealed that it was difficult to achieve it, as various factors, such as social context and surroundings could influence this process (Lyons et al., 2014).

Knowing when to stop drinking is based on experience and monitoring physical changes, rather than on factors such as counting units. Some men described this knowledge as being ‘subconscious’, and attributed it to being an ‘experienced’ drinker able to control their alcohol consumption (Lyons et al., 2014). Others relied on bodily cues and stopped drinking after experiencing negative physical effects, such as dizziness or being too loud. This state was often reached suddenly and unexpectedly (Lyons et al., 2014). ‘Passing the point of no return’ relates to a sudden realisation that too much alcohol has been consumed and is closely linked to the previous sub-theme of loss of control. This feeling was associated with an altered physical stance or social context. For example, participants described realising they had drunk too much only when they got up from their seat, or moved outside after drinking indoors (Lyons et al., 2014).

Midlife men expressed feelings of superiority, viewing themselves as being more experienced drinkers, able to drink more and be in control of their behaviour, as opposed to younger men who might find it hard to ‘keep their cool’ (Rolfe et al., 2006). This links directly to the ‘tolerance’ justification mentioned above.

However, having reached an optimal state of intoxication, some participants described that they continued to drink and attributed it to loss of control, as they felt that they could not stop despite experiencing various negative effects. The participants used metaphors to describe the powerful effect of alcohol on their behaviour as they ‘went with the flow’ and ‘got bladdered’ without wanting to do so (Brierley-Jones et al., 2014; Emslie et al., 2012). Furthermore, social aspects also contributed to ‘losing control’ due to influences such as peer pressure (e.g., buying rounds (Emslie et al., 2013)). Drinking in a group could involve ‘getting carried away’ and lead to excessive consumption of alcohol (Foster & Heyman, 2013; Lyons et al., 2014). Some participants blamed the loss of control as leading to engaging in behaviours that they would not do had they been sober and described feeling embarrassed and regretful after doing so (e.g., going home with a stranger (Lyons et al., 2014; Orford et al., 2002)).

### 3.6 Social norms and identity

Drinking behaviours were guided by strong social norms existing within social groups and drinking alcohol linked to specific identities. There are two key sub-themes to consider: alcohol as a symbol of masculinity; and social judgements on price and quality of alcohol.

Strong associations were identified between masculinity and specific aspects of the purchasing and consumption of alcohol. Drinking pints of beer in the pub with friends was widely recognised to be a masculine activity – one which was highly valued (Brierley-Jones et al., 2014; Emslie et al., 2012; Emslie et al., 2013; Lyons et al., 2014; Wilson et al., 2013). Drinking something other than beer or lager in these settings could cause others within the social group to question one's masculinity (Emslie et al., 2012; Emslie et al., 2013). Wine drinking could also have masculine associations, although not usually within pub settings. Instead, knowledge of wine could demonstrate masculinity when choosing a fine wine at a restaurant, or selecting a bottle for a specific meal or occasion (Emslie et al., 2013; Ritchie, 2007). In male-female couples, these were all tasks that the male was more likely to perform (Ritchie, 2007).

The selection of a certain type of drink could influence an individual's sense of identity and self-esteem. Wine was described as more ‘classy’ or ‘sophisticated’ by some (Brierley-Jones et al., 2014; Ritchie, 2007), and expensive wines could be used to demonstrate ‘cultural and financial exclusivity’ (Ritchie, 2007). Some men expressed dissatisfaction with drinking stronger, cheaper beers or ciders during periods of unemployment, and the satisfaction they experienced when they were able to afford more premium, branded beers in the pub (Emslie et al., 2013).

### 3.7 Harm

The studies illuminated perceived and experienced harm associated with alcohol consumption in midlife, beliefs surrounding the drinking guidelines and views about moderation and reduction of consumption. The following sub-themes were identified: negative psychological and physical effects of drinking; concerns surrounding male mental health and emotional vulnerability; link between alcohol and aggression; interpretation of guidelines; rejection of harmful effects on health; reasons for reducing consumption and perceived drawbacks and benefits of drinking.

Participants had experienced various negative psychological effects of excessive drinking, such as exacerbation of negative mood states, humiliation (Orford et al., 2002), uncontrollable aggression, irritability (Rolfe et al., 2006), as well as guilt and shame caused by secretive drinking (Wilson et al., 2013). Drinking was described as having a negative impact on physical wellbeing, with some participants describing that as they had got older they experienced more severe hangovers (e.g. stronger headaches) lower tolerance and higher unpredictability of the effects of alcohol in comparison with when they were younger, which made some of them limit their drinking (Lyons et al., 2014). So while many older drinkers described themselves as ‘experienced drinkers’, some also identified a reduced tolerance to alcohol. Some older drinkers found it harder to recover after a ‘heavy session’ (Emslie et al., 2012) and described being ‘punished’ the following day due to the social responsibilities which still needed to be met while feeling hung-over (Lyons et al., 2014; Orford et al., 2002). In addition, some participants reported negative effects of excessive drinking on eating behaviour and sexual performance (Orford et al., 2002).

Some men described ‘session depression’ and ‘the Sunday blues’, and exacerbated negative mood (e.g. guilt) following a heavy drinking session (Emslie et al., 2013). In the same study, men explained that they relied on alcohol as a tool enabling them to express their emotions, in contrast with women who they saw as more socially competent (Emslie et al., 2013).

The link between alcohol and aggression was also identified in some studies. Alcohol was identified as a causal factor (‘like a fuse…waiting to go off’ (Rolfe et al., 2006)) in releasing aggressive impulsive behaviour and violence (‘bravado’, ‘being lairy’), and this was perceived to be the case especially in younger men (Brierley-Jones et al., 2014), who they saw as in contrast to themselves, being older and ‘more sensible’ (Orford et al., 2009). This however was not always evident, as some midlife male participants saw alcohol-induced fighting as a symbol of masculinity (Rolfe et al., 2006), although the majority of the participants distanced themselves from such behaviour by describing it as being ‘in the past’, despite alcohol also being described as a contributing factor in cases of domestic violence (Rolfe et al., 2006).

Participants expressed cynicism and rejection of the notion of units (Brierley-Jones et al., 2014) and guideline recommendations, describing them as ‘shifting sands’ and being ‘abstract’ or ‘arbitrary’ (Lyons et al., 2014). Some midlife men justified their excessive drinking by describing how despite regularly consuming more than their recommended limit, they were still able to be in control of their behaviour (Wilson et al., 2013). Related to this, participants generally were sceptical about statements on the harmful effects of alcohol on health, and any awareness they had of the possible harmful effects was likely to be kept ‘in the back of their mind’ (Orford et al., 2002; Wilson et al., 2013). Although participants suffering serious health conditions did identify the harmful effect of excessive drinking alongside medication, a fatalistic attitude to continued drinking was often expressed (Wilson et al., 2013).

The reasons midlife drinkers described for reducing their alcohol consumption included health concerns, such as a recent hospitalisation and ageing (Brierley-Jones et al., 2014; Lyons et al., 2014); realisation of own excessive drinking by comparing oneself to other drinkers and the risk of becoming an alcoholic (Foster & Heyman, 2013); and different priorities related to social events, such as becoming parents, moving and changing jobs/social circles (Emslie et al., 2012).

A final sub-theme captures a sense by participants that the drawbacks of drinking are outweighed by the benefits, which may to some extent explain the maintenance and normalisation of heavy drinking in midlife (Orford et al., 2002).

## 4 Discussion

The qualitative studies reviewed in this paper highlight the significance of the meanings and social importance of alcohol consumption to midlife men in the UK, consistent with wider research on how social and cultural factors influence the specific pattern of alcohol consumption within a society (Castro et al., 2014). The review suggests strong motivations for midlife men to drink: they do it to relax as part of an everyday routine and they do it as an important part of socialising with others, particularly other men. Indeed, some midlife men feel that without the drinking it would be difficult, if not impossible, to have any meaningful social interaction with other men in their free time. Coupled with this, midlife men generally state that their drinking is un-problematic: they believe to be in control of it and that it does not interfere with their daily responsibilities. This is in agreement with other research on UK men's drinking cultures and self-perceptions (Thurnell-Read, 2017). To support this perception, the men have a strong idea about what a real ‘problem drinker’ looks like, and this ‘othering’ appears to work not only by stigmatising this distant, ‘not-me’ problem drinker, but perhaps more importantly by protecting the men from accusations (by others) and self-perceptions of having a problematic relationship with alcohol. For the men there was a clear association between their drinking, mental wellbeing and motivations for drinking, as found also in other research (Appleton, James, & Larsen, 2018).

The midlife men in the reviewed studies use elaborate strategies to manage their drinking to maximise the beneficial experiential and social effects; but while these for some involve clear boundaries regulating when to stop drinking to avoid negative experiential and social effects, for others (or at other times), the perception of their own ability to control their drinking is not as pronounced. A consolation for the midlife men is, however, that they generally feel that they now are much better able to control this compared to when they were younger. The men feel that they strongly benefit from their identity as ‘drinkers’ in terms of the cultural norms of masculinity and strength it is associated with, as found in other research (De Visser & Smith, 2007; Thurnell-Read, 2017). The male status is seen as associated with being able to drink large quantities, drinking particular alcoholic drinks as well as having expert knowledge of alcoholic drinks and performing social roles related to ordering and buying drinks.

The role of drinking, and drinking particular types of drinks, as affirmation of socially constructed notions of masculinity (Bridges & Pascoe, 2014; Connell, 1995) resonates with findings internationally. For example, public drinking in Vietnam has been described as ‘encouraging a masculinized form of binge drinking’ (Gillen, 2016), and in New Zealand beer advertising offers a ‘nostalgic valorisation of a local hegemonic masculinity in a time of destabilised male identity politics’ (Gee & Jackson, 2012). Although it is important to not essentialise ‘male drinking’ as a uniform practice, which it is clearly not, these findings suggest that drinking practices can, at least in part, be understood as a way for some men in different cultural contexts to demonstrate idealised forms of masculinity to assume positions of power. Courtenay has argued that by taking greater health risks men legitimise themselves as the ‘stronger’ sex, and that this helps to sustain and reproduce social inequality and the social structures that, in turn, reinforce and reward men's poor health habits (Courtenay, 2000). Hence, men's perceived or culturally ingrained benefits of established risky drinking practices may be considerable.

While the evidence reviewed suggests that UK midlife men have some knowledge of the harms that can be caused by drinking alcohol, it is mainly short-term harms (such as hangovers and effects on mood) they are concerned about, and they mainly associate longer-term health harms with the abovementioned ‘problem drinker’. In agreement with recent research on attitudes to alcohol guidelines (Lovatt et al., 2015), the review identified general scepticism in respect to the value and personal relevance of such guidance. Midlife men believe that they can base the judgement of whether their drinking is a problem or not on how they feel about it themselves, feeling in control and meeting personal responsibilities in terms of work, children and driving. The data suggest that the men might consider moderating their drinking if they experienced major health concerns, if they felt their drinking was excessive when compared to others and if changed social circumstances required reduced drinking. However, further detail on this and how such messages could be communicated would require further investigation.

### 4.1 Study limitations

Only one of the included papers drew on data exclusively from midlife men, which could mean that insights pertaining specifically to these were diluted. Furthermore, the findings highlighted here relate to a group of middle-aged men from a diversity of geographical regions within the UK. Participants were also diverse with regards to their levels of alcohol consumption, spanning from low risk or ‘occasional’ drinkers, to those classified as harmful or dependent drinkers. Information regarding socioeconomic status and ethnicity were less well reported by primary study authors, but there is indication that some study authors did attempt to achieve diversity in these respects. The extensive reliance on focus groups is a limitation as this methodology, while good at exploring social norms, is less useful for revealing individual motivations and experiences. A limitation of the thematic synthesis approach is that it does not allow investigation of the richness of the original full qualitative data sets, but accesses the insights through the analytical perspectives and illustrative data selections applied by the authors of the original studies. However, this third level interpretation (Pearce et al., 2015) identified significant common observations and themes, which does offer some reassurance of the validity of these. We recommend further in-depth research in the UK focussing exclusively on midlife men and that future work in this field ensures clear reporting of participant demographics, including socioeconomic status, ethnicity and family structure. Finally, although some findings from this review of UK research do appear to resonate with other research on male drinking internationally, it would not be possible to generalise details of the insights outside a UK context, and reviews of these other culturally-specific bodies of research would be welcomed.

## 5 Conclusions

The evidence of increasing harm experienced among midlife men in the UK as a result of excessive drinking provides a strong rationale for research exploring the experiences of and motivations for drinking in this group. The findings presented in this review make it clear that any intervention built around information and guidance aiming to effectively influence the way these men drink would have to carefully consider insights concerning the cultural meanings and social importance they associate with their alcohol consumption.

The following are the supplementary data related to this article.

Supporting information 1 Full Medline search strategy. Supporting information 1

Supporting information 2 CASP quality appraisal tool for qualitative research (adapted).* Supporting information 2

Supporting information 3 Data extraction fields. Supporting information 3

Supporting information 4 Why the primary study is relevant to a review of drinking in middle-aged men. Supporting information 4

Supporting information 5 Detailed CASP quality results. Supporting information 5

Supporting information 6 Illustrative quotes. Supporting information 6

## Author statements

No research ethics approval was required for this review study as it did not involve the collection of new empirical data. No dedicated research funding was received as the authors did the work as part of their day job research roles: three as employees of academic institutions (CH, JL, AM), one as a student placement (MM), and three (HP, AR, JLa) as employees in the research function of the UK-wide alcohol education charity Drinkaware. The charity is established through an agreement between the UK government and the alcohol industry, and is funded primarily by voluntary and unrestricted donations from UK alcohol producers, retailers and supermarkets. Drinkaware is governed independently and works in partnership with others to reduce alcohol-related harm by helping people make better choices about their drinking.
