# Supplementary material for: Understanding drinking among midlife men in the United Kingdom: A systematic review of qualitative studies
Source: Addict Behav Rep. 2018 Aug 4;8:85–94. doi: 10.1016/j.abrep.2018.08.001 (PMC6104518; doi:10.1016/j.abrep.2018.08.001)
Supplement: Supporting information 1 — Full Medline search strategy. [file mmc1.docx]

**Supporting Information 1**

Full Medline Search Strategy

1. Exp Alcohol drinking/
2. Exp Alcoholism/
3. Exp Alcoholic intoxication
4. Alcohol*.mp
5. Drink*.mp
6. Drunk*.mp
7. Booz*.mp
8. Or/1-7
9. Exp Attitude/
10. Exp Attitude to health/
11. Belie*.mp
12. Attitud*.mp
13. Behav*.mp
14. View*.mp
15. Motivat*.mp
16. Or/9-15
17. Exp United Kingdom/
18. UK.mp
19. “United Kingdom”.mp
20. GB.mp
21. Brit*.mp
22. Engl*.mp
23. Scot.mp*
24. Northern Ir*.mp
25. Wales.mp
26. Welsh.mp
27. Or/17-27
28. 8 and 16 and 27
