# Supplementary material for: Understanding drinking among midlife men in the United Kingdom: A systematic review of qualitative studies
Source: Addict Behav Rep. 2018 Aug 4;8:85–94. doi: 10.1016/j.abrep.2018.08.001 (PMC6104518; doi:10.1016/j.abrep.2018.08.001)
Supplement: Supporting information 2 — CASP quality appraisal tool for qualitative research (adapted).* [file mmc2.docx]

**Supporting information 2**

**CASP Quality Appraisal Tool for Qualitative Research (adapted) ^*^**

Score 2 if fully addressed; 1 if partially addressed; 0 if not addressed at all

| 1. **Was there a clear statement of the aims?**   What was the goal of the research  Why it was thought important  Its relevance |
| --- |
| 1. **Is a qualitative methodology appropriate?**   If the research seeks to interpret or illuminate the actions and/or subjective experiences of research participants  Is qualitative research the right methodology for addressing the research goal |
| 1. **Was the research design appropriate to address the aims of the research?**   If the researcher has justified the research design |
| 1. **Was the recruitment strategy appropriate to the aims of the research?**   If the researcher has explained how the participants were selected  If they explained why the participants they selected were the most appropriate to provide access to the type of knowledge sought by the study  If there are any discussions around recruitment |
| 1. **Was the data collected in a way that addressed the research issue?**   If the setting for data collection was justified  If it is clear how data were collected  If the researcher has justified the methods chosen  If the researcher has made the methods explicit  If methods were modified during the study. If so, has the researcher explained how and why?  If the form of data is clear  If the researcher has discussed saturation of data |
| 1. **Has the relationship between researcher and participants been adequately considered?**   If the researcher critically examined their own role, potential bias and influence during (a) Formulation of the research questions (b) Data collection, including sample recruitment and choice of location  How the researcher responded to events during the study and whether they considered the implications of any changes in the research design |
| 1. **Have ethical issues been taken into consideration?**   If there are sufficient details of how the research was explained to participants for the reader to assess whether ethical standards were maintained  If the researcher has discussed issues raised by the study  If approval has been sought from the ethics committee |
| 1. **Was the data analysis sufficiently rigorous?**   If there is an in-depth description of the analysis process  If thematic analysis is used. If so, is it clear how the categories/themes were derived from the data?  Whether the researcher explains how the data presented were selected from the original sample  If sufficient data are presented to support the findings  To what extent contradictory data are taken into account  Whether the researcher critically examined their own role, potential bias and influence during analysis and selection of data for presentation |
| 1. **Is there a clear statement of findings?**   If the findings are explicit  If there is adequate discussion of the evidence both for and against the researchers arguments  If the researcher has discussed the credibility of their findings  If the findings are discussed in relation to the original research question |
| 1. **How valuable is the research?**   If the researcher discusses the contribution the study makes to existing knowledge or understanding  If they identify new areas where research is necessary  If the researchers have discussed whether or how the findings can be transferred to other populations or considered other ways the research may be used |

* Critical Appraisal Skills Programme (2013) CASP Qualitative Checklist. [Online] Available at: <http://www.casp-uk.net/#!casp-tools-checklists/c18f8>
