# Supplementary material for: Understanding drinking among midlife men in the United Kingdom: A systematic review of qualitative studies
Source: Addict Behav Rep. 2018 Aug 4;8:85–94. doi: 10.1016/j.abrep.2018.08.001 (PMC6104518; doi:10.1016/j.abrep.2018.08.001)
Supplement: Supporting information 3 — Data extraction fields. [file mmc3.docx]

**Supporting Information 3**

**Data Extraction Fields**

| Title |
| --- |
| Authors |
| Links to other studies? |
| Publication Date (YYYY) |
| Journal |
| Study Aim |
| Population |
| Methods of data collection |
| Period of data collection |
| Method of data analysis |
| Findings (copy and paste directly everything labelled 'results' or 'finding' including participant quotes) |
| Discussion and conclusions (summary only- use abstract plus anything else you think is important from the full text) |
| Limitations and weaknesses (as noted by the authors) |
| Any additional notes or comments from reviewer |
