# Supplementary material for: Understanding drinking among midlife men in the United Kingdom: A systematic review of qualitative studies
Source: Addict Behav Rep. 2018 Aug 4;8:85–94. doi: 10.1016/j.abrep.2018.08.001 (PMC6104518; doi:10.1016/j.abrep.2018.08.001)
Supplement: Supporting information 4 — Why the primary study is relevant to a review of drinking in middle-aged men. [file mmc4.docx]

**Supporting information 4 – Why the primary study is relevant to a review of drinking in middle-aged men**

| **Review** | **Illustrative quotes from primary study authors distinguishing findings relevant to middle aged men** |
| --- | --- |
| Brierley-Jones et al (2014) | - “Most quotations illustrating the home drinking habitus were made by women in FG 4. Most of the quotations illustrating the traditional drinking habitus were made by men in FG 3.” |
| Emslie, Hunt & Lyons (2012)  [DrAM 1 of 3] | - “In terms of gender, both men and women saw drinking as pleasurable and a fun way to socialize.” - “Both men and women experienced pressure to drink and referred to alcohol both as a reward and a way to recover from – or survive – the rigours of paid work, unpaid work in the case of women, and other stresses.” - “Male participants were able to state that they were going out with the intention of getting drunk (albeit while still demonstrating they could fulfil their responsibilities).” |
| Emslie, Hunt & Lyons (2013)  [DrAM 2 of 3] | - “We focus solely on the male respondents to explore how alcohol is associated with “doing” masculinity in midlife.” |
| Lyons, Emslie & Hunt (2014)  [DrAM 3 of 3] | - “We chose to conduct same-sex and mixed-sex groups…to explore similarities and differences in gendered, embodied experiences across different groups. “ - “This was gendered due to the differences in everyday social roles. Generally, for men, drinking alcohol provided embodied pleasure as a reward for working hard” - “Both men and women described this state of enjoyment as being in the zone or that perfect level.” |
| Foster, Read, Karunthi et al. (2010)  [1 of 2] | - “A summary of the themes and the gender and age distribution of the comments are shown in Table 1.” - “The theme of convenience was common for both genders and for individuals aged 16–55.” - “Cost was discussed most frequently by males and individuals aged 26–35 and 46–55.” |
| Foster & Heyman (2013)  [2 of 2] | - “They all saw themselves as being ‘sensible’ drinkers.” - “Group A, which included the youngest participants, tended to describe the most risky behaviours; and Group C, individuals recruited through a residents’ association of a housing estate were the most risk-averse.” |
| Orford, Dalton, Hartney et al. (2002)  [BUHD 1 of 3] | - *Presents a model using findings generalisable across population sample:* “The aim was then to develop a model of how heavy drinking might be maintained in the face of the reported benefits and drawbacks.” |
| Rolfe, Dalton, Krishnan et al. (2006)  [BUHD 2 of 3] | - “The analysis for the present paper is based on qualitative data on the topics [including]… masculinity and alcohol (men only, n=23)” - “Men also talked about fighting as part of being a man and particularly as part of a macho culture. It was a commonly expressed view that men often went drinking looking for a fight or expecting trouble.” |
| Orford, Rolfe, Dalton et al. (2009)  [BUHD 3 of 3] | - “Because the views of men were over-represented and gender has not been considered in the present analysis (data from the same study are being considered from a gender perspective elsewhere), it is also possible that the conclusions drawn may represent a male-biased perspective, particularly if the pub continues to be an arena of special importance for the expression of forms of masculinity.” |
| Ritchie (2007) | - “In a partnership most of the overt, and almost all the specialist, wine purchasing was performed or believed to be performed by the male partner…overt wine buying was perceived as a masculine activity.” - “All participants agreed that they bought and consumed differently according to mood. “ |
| Wilson, Kaner, Crosland et al. (2013) | - “Whilst specific references to gender were infrequent, the fact that they were evident at all suggests that older adults recognised gender-based patterns of drinking and also issues of stigma in relation to public drinking by women and deviations from male drinking stereotypes, which could challenge attempts to reduce consumption, such as drinking smaller quantities of alcohol. “ |
