# Supplementary material for: Understanding drinking among midlife men in the United Kingdom: A systematic review of qualitative studies
Source: Addict Behav Rep. 2018 Aug 4;8:85–94. doi: 10.1016/j.abrep.2018.08.001 (PMC6104518; doi:10.1016/j.abrep.2018.08.001)
Supplement: Supporting information 5 — Detailed CASP quality results. [file mmc5.docx]

**Supporting Information 5**

Detailed CASP quality results

| CASP Criteria | Brierley-Jones, 2014 | Emslie, 2012 | Emslie, 2013 | Lyons, 2014 | Foster, 2010 | Foster, 2013 | Orford, 2002 | Rolfe,  2006 | Orford, 2009 | Ritchie, 2007 | Wilson, 2013 |
| --- | --- | --- | --- | --- | --- | --- | --- | --- | --- | --- | --- |
| **Was there a clear statement of the aims?** | 2 | 2 | 2 | 2 | 2 | 2 | 2 | 2 | 2 | 2 | 2 |
| **Is a qualitative methodology appropriate?** | 2 | 2 | 2 | 2 | 2 | 2 | 2 | 2 | 2 | 2 | 2 |
| **Was the research design appropriate to address the aims of the research?** | 1 | 1 | 1 | 1 | 1 | 1 | 1 | 1 | 2 | 1 | 2 |
| **Was the recruitment strategy appropriate to the aims of the research?** | 2 | 2 | 1 | 1 | 1 | 1 | 2 | 1 | 2 | 2 | 2 |
| **Was the data collected in a way that addressed the research issue?** | 2 | 2 | 1 | 1 | 1 | 1 | 2 | 2 | 2 | 2 | 2 |
| **Has the relationship between researcher and participants been adequately considered?** | 0 | 0 | 1 | 0 | 1 | 0 | 1 | 0 | 0 | 0 | 0 |
| **Have ethical issues been taken into consideration?** | 2 | 2 | 2 | 2 | 1 | 1 | 0 | 2 | 0 | 0 | 2 |
| **Was the data analysis sufficiently rigorous?** | 1 | 1 | 1 | 1 | 1 | 1 | 1 | 1 | 2 | 0 | 2 |
| **Is there a clear statement of findings?** | 2 | 2 | 2 | 2 | 2 | 2 | 2 | 2 | 2 | 2 | 2 |
| **How valuable is the research?** | 2 | 2 | 2 | 2 | 2 | 2 | 2 | 1 | 1 | 2 | 2 |
| **Total score / 20** | **16** | **16** | **15** | **14** | **14** | **13** | **15** | **14** | **15** | **13** | **18** |
