# Supplementary material for: Understanding drinking among midlife men in the United Kingdom: A systematic review of qualitative studies
Source: Addict Behav Rep. 2018 Aug 4;8:85–94. doi: 10.1016/j.abrep.2018.08.001 (PMC6104518; doi:10.1016/j.abrep.2018.08.001)
Supplement: Supporting information 6 — Illustrative quotes. [file mmc6.docx]

**Supporting Information 6**

**Illustrative Quotes**

| **Theme** | **Sub-theme** | **Illustrative quotes**  **(Text in italics comes directly from study participants, non-italic text comes directly from study authors)** |
| --- | --- | --- |
| Drinking Motivations | Drinking to relax and its’ distinction from drinking to cope | - “Wine was drunk in many cases, not to relieve stress but for the pleasure it gave: thus, in response to the query, ‘*do you drink at home for … the stress thing, or do you enjoy it?*’ the answer was ‘*I just enjoy it.*’ (Male, home drinker).” Brierley-Jones et al., 2014 - “A separate stage of problematic drinking, in contrast, involved an escalation to more and stronger alcohol than he had drunk previously in order to cope” Wilson et al., 2013 |
|  | Drinking to socialise and its’ importance in establishing and maintaining friendships | - “Going out drinking together was widely constructed as the “*natural*” way for men to socialize and to enact their ties of (male) friendship” Emslie, Hunt & Lyons 2013 - “Often referred to was [the pub’s] function as a place where relationships were maintained, where you ‘*bump into’*, ‘*catch up with*’ or ‘*swap gossip with*’ friends.” Orford et al., 2009 |
|  | Drinking to get drunk | - “Drinking beer, lagers and spirits was associated with having ‘*belly laughs and the hilarity*’, of having a ‘*mad*’ or ‘*daft*’ night. Some reported ‘*losing control*’ and sought to ‘*get like whoohoo*’ when out drinking.” Brierley-Jones et al., 2014 - “Some participants said they did not drink alcohol to relax or escape, but to binge drink and get drunk” Lyons, Emslie & Hunt 2014 |
| Drinking Justifications | Drinking as a controlled choice | - “In positive terms, alcohol was treated as something that could be enjoyed by older people who consumed it minimally or occasionally. Alcohol was drunk through choice, and therefore implied control over one’s actions” Wilson et al., 2013 |
|  | Meeting responsibilities | - “A central theme was that as respondents entered mid-life, they had to plan their drinking around their ‘responsibilities’ (particularly children, paid work and driving). Self control and discipline were necessary in order to prioritise responsibilities and counteract the powerful appeal of alcohol.” Emslie, Hunt & Lyons 2012 - “*‘I have a nine-year old son, and we don’t make a habit of having a drink when he is up. So if he goes to bed at nine o’clock, then we will have a drink. It is not reasonable to drink at five or six o’clock’*” (Male, 35yo, community volunteers focus group) Foster & Heyman 2013 |
|  | Othering of problematic drinking | - “The typical home drinker perceived regular, moderate home drinking of wine as unproblematic when compared with the large quantities consumed in the social, weekend drinking of beer, lager and spirits associated with the traditional drinker…Traditional drinkers, too, were keen to distinguish their controlled drinking pattern from the vulgar excesses of the ‘problem drinker’.” Brierley-Jones et al., 2014 - “Youthful drinking was associated with aiming to become drunk quickly (and so choice of drink was inﬂuenced by the price and strength of alcohol content), whereas drinking in early mid-life was characterised as being relaxing, sociable and civilised.” Emslie, Hunt & Lyons 2012 |
| Drinking Strategies and Control | Drinking strategies | - *“ ‘Subconsciously as well, I would always, if I knew I was going out for a fairly big night out, have soup. This sounds really, really grim, like it sounds terrible, but what I’m thinking is, ‘What if I get so drunk that I’m sick, or even sick in my sleep. If I’m being sick liquid, I’ll survive’. [Laughter]. But I think if I have a big meal, I’m far more likely to be sick, and also if I’m sick and it’s just soup at least it’s liquid coming out, if it’s horrible big, you know, meat or whatever, then it’s going to be fatal.’ ”* (Male, unemployed, 28-31yo). Lyons, Emslie & Hunt 2014 |
|  | Being ‘in the zone’ | - *“ ‘I’d love to drink all night but not get drunk. Get to that level of – that perfect level, you know, and stay there … in the zone, that’s what it is.’ “* (Male, lecturer, 34 -49 yo) Lyons, Emslie & Hunt 2014 |
|  | Knowing when to stop | - “When limiting their consumption participants did not count units … but instead monitored their changing embodied states… Alongside achieving this enjoyable state, the participants talked about reaching a tipping point or a point of no return…when they realised they had consumed too much alcohol and were feeling less pleasant sensations.” Lyons, Emslie & Hunt 2014 |
|  | Drinking more with age and feeling superior to younger men | - “[One participant] presents the drinking of 10 pints by a drinking companion in one session as controlled and occasional... This is finally reinforced by aligning his own drinking with that of his companion and treating this as humorous, then suggesting that only younger people without their experience are likely to lose self-control after such consumption.” Wilson et al., 2013 |
|  | Loss of control | - Self-control and discipline were necessary in order to prioritise responsibilities and counteract the powerful appeal of alcohol, often referred to using metaphors suggesting the natural force of water (e.g. ‘*swept along’, ‘go with the ﬂow’*). Emslie, Hunt & Lyons 2012 - “Several people referred to their judgment being adversely affected after drinking. ‘‘*Brain’s out, beer takes over’’* as one participant put it.” Orford et al., 2002 |
|  | Passing the ‘point of no return’ | - “Changing physical stance and/or moving away from the immediate social situation (for example, going to the toilet or standing up) was frequently the ﬁrst moment when participants realised that they had passed that limit.” Lyons, Emslie & Hunt 2014 |
| Social Norms and Identity | Alcohol as a symbol of masculinity | - “Respondents in most of the heavier drinking groups found the idea of men going out for coffee or a meal together, rather than a pint, unlikely or laughable.” Emslie, Hunt & Lyons 2012 - “Men’s heavy consumption of beer with groups of other men in pubs was valorised” Wilson et al., 2013 |
|  | Social judgements on price and quality of alcohol | - “Participant perceptions were that wine was ‘classy’ – more sophisticated than other alcoholic drinks.” Ritchie 2007 - “Unemployed men constructed their current position (drinking strong, cheap cider or beer at home) as stigmatized, and contrasted this with the satisfaction they felt when they were working and able to afford to drink the leading Scottish brand of beer in the pub.” Emslie, Hunt & Lyons 2013 |
| Harm | Negative psychological and physical effects of drinking | - “A minority described very unpleasant after-effects [of drinking], including depression and ‘‘despair’’, getting the shakes and wanting to ‘‘hibernate’’ and not leave the house, or hangovers becoming worse with age to the point of feeling close to death on occasions.” Orford et al., 2002 |
|  | Concerns surrounding male mental health and emotional vulnerability | - “Respondents were aware that alcohol is a depressant and its aftereffects were referred to colloquially as ‘*session depression*’ and ‘*the Sunday Blues*’.” Emslie, Hunt & Lyons 2013 |
|  | Link between alcohol and aggression | - “It was a commonly expressed view that men often went drinking looking for a fight or expecting trouble.” Rolfe, et al., 2006 |
|  | Interpretation of guidelines | - “Participants reached a consensus regarding scepticism about medical advice provided on alcohol. It could be agreed, for instance, that health professionals were overly ready to attribute older people’s health problems to their alcohol intake.” Wilson et al., 2013 |
|  | Rejection of harmful effects on health | - “Most focus group members did not believe that their alcohol consumption was causing risks to their future health, as they saw their drinking as merely episodic.” Foster & Heyman 2013 - “What was very noticeable was the sparseness of references, in the open-ended material, to concern about the possible chronic effects of alcohol intake on physical health.” Orford et al., 2002 |
|  | Reasons for reducing consumption | - “Both fathers and mothers in the sample felt that their drinking had reduced when their children were very young – particularly because of the difﬁculty of getting up early to look after young children after a heavy night’s drinking.” Emslie, Hunt & Lyons 2012 - “Some participants, especially in the residents’ focus group, indicated that in the past they had been concerned about risks of drinking adversely affecting their future health. They tended to see their high level of alcohol consumption as caused by others, usually partners or friends, and had implemented life changes as a way of minimising the consequent risks.” Foster & Heyman 2013 |
|  | Perceived drawbacks and benefits of drinking | - “Even amongst this group of very heavy drinkers, the perceived benefits of drinking outweigh the drawbacks. This was true for the sample as a whole, for men and for women, and for the large majority of individual members of the sample.” Orford et al., 2002 |
